# ARID1A in cancer: Friend or foe?

**DOI:** 10.3389/fonc.2023.1136248

**Published:** 2023-02-20

**Authors:** Beatrice Fontana, Giulia Gallerani, Irene Salamon, Ilaria Pace, Roberta Roncarati, Manuela Ferracin

**Affiliations:** ^1^ Department of Medical and Surgical Sciences (DIMEC), University of Bologna, Bologna, Italy; ^2^ Istituto di Genetica Molecolare ”Luigi Luca Cavalli-Sforza“ – Consiglio Nazionale delle Ricerce (CNR), Bologna, Italy; ^3^ IRCCS Azienda Ospedaliero-Universitaria di Bologna, Bologna, Italy

**Keywords:** ARID1A, tumor suppressor gene, oncogene, solid tumors, SWI/SNF complex, synthetic lethality

## Abstract

ARID1A belongs to a class of chromatin regulatory proteins that function by maintaining accessibility at most promoters and enhancers, thereby regulating gene expression. The high frequency of ARID1A alterations in human cancers has highlighted its significance in tumorigenesis. The precise role of ARID1A in cancer is highly variable since ARID1A alterations can have a tumor suppressive or oncogenic role, depending on the tumor type and context. ARID1A is mutated in about 10% of all tumor types including endometrial, bladder, gastric, liver, biliopancreatic cancer, some ovarian cancer subtypes, and the extremely aggressive cancers of unknown primary. Its loss is generally associated with disease progression more often than onset. In some cancers, ARID1A loss is associated with worse prognostic features, thus supporting a major tumor suppressive role. However, some exceptions have been reported. Thus, the association of ARID1A genetic alterations with patient prognosis is controversial. However, ARID1A loss of function is considered conducive for the use of inhibitory drugs which are based on synthetic lethality mechanisms. In this review we summarize the current knowledge on the role of ARID1A as tumor suppressor or oncogene in different tumor types and discuss the strategies for treating ARID1A mutated cancers.

## Introduction

### Physiological role of ARID1A

Nucleosomes are composed of 147 base-pairs of DNA wrapped around histone octamers (1) and constitute the core unit of chromatin, which is further organized and compacted in higher order structures called topologically associated domains (TADs) in the nucleus (2, 3). The chromatin needs to be dynamically remodeled to guarantee the activation or repression of gene expression during the entire life of the cell, but particularly during the embryonic development (4) and differentiation (5). Remodeling means that regulatory complexes can be “opened” to provide access to the underlying DNA to enable transcription, chromatin assembly, DNA repair, and other processes. Chromatin remodeling is altered in pathological conditions such as cancer and heart failure (6, 7). Different classes of histone modifying enzymes that are involved in the deposition of histone tail modifications, including acetylation, methylation, phosphorylation, SUMOylation and ubiquitination, were identified. Furthermore, there are other ATP-dependent chromatin remodeling complexes, like the SWI/SNF complex, which uses the energy produced from ATP hydrolysis to mobilize and modify the nucleosome chromatin block and recruit the transcriptional machinery to the DNA (8).

The SWI/SNF complex was identified in yeast in 1984 (9) and it was later demonstrated that its structure is highly conserved among species, including mammals (10), suggesting a functional conservation during evolution. The mammalian SWI/SNF complex is comprised of more than 15 subunits (encoded by 29 genes) which assemble into three different complexes: BRG1/BRM-associated factor complex (BAF), polybromo-associated BAF complex (PBAF), and non-canonical BAF (ncBAF) (11). The AT-rich interacting domain containing respectively protein 1A (ARID1A) and 1B (ARID1B) subunits belong to the canonical BAF complex. The main role of ARID1A and B is to link the BAF core module to the subunits with ATPase activity (11). Both proteins are expressed in mammalian cells, and show a specific localization map onto the genome, characterized by a mutually exclusive interaction with the SWI/SNF complex (12).

ARID1A (also known as BAF250a, p270 or SMARCF1) is a key component of the mammalian SWI/SNF protein complex. ARID1A directly binds DNA with low sequence specificity (13), even though recently Rahmanto et al. described some specific DNA binding motifs which were found enriched in ARID1A ChIP-seq peaks in endometrial tumor cells (14). This observation suggests the formation of specific co-regulatory modules in which SWI/SNF complex, and particularly ARID1A subunit, co-localize with different transcription factors (AP-1 (15), FOXA1(16)) to regulate gene transcription in a cell-type specific manner. Moreover, ATAC-seq experiments conducted in colon cancer HCT116 cells proved that the loss of ARID1A profoundly altered chromatin accessibility, revealing a pivotal role of ARID1A in chromatin organization, determining a “gain or loss” of accessibility (15). Kelso et al. also demonstrated that the loss of ARID1 primarily affects enhancer accessibility and active histone marks on these regulatory regions, resulting in a significant alteration of the overall gene expression. Furthermore, ARID1A regulates transcription by modulating the conservative mechanism of RNA polymerase II (RNAPII) pausing which allows rapid and efficient transcription of several loci. This is a mechanism required to maintain cell homeostasis and development (17, 18). The analysis of RNAPII dynamics in ovarian epithelial cells revealed that loss of ARID1A induce changes in pausing versus elongating RNAPII fraction, leading to a significant reduction of transcription on active genes (19). Gao et al. demonstrated in mice that *ARID1A* deletion on one allele leads to embryonic lethality (20).

In addition, ARID1A can modulate gene transcription in cells, either directly controlling the expression of cancer related genes or indirectly by regulating the recruitment or activity of histone modifier enzymes, which add or remove histone modifications at gene regulatory regions. For example, ARID1A can regulate the immune response by modulating the interferon-responsive gene methylation profile in multiple tumor types (21). ARID1A is also involved in the regulation of Topoisomerase IIa (TOP2A) recruitment on chromatin, which is necessary to resolve R-loop formation during transcription (22).

The immunoprecipitation assay for ATR (ataxia telangiectasia and RAD3-related protein) followed by mass spectrometry, revealed that ARID1A is also an interacting partner and enriched in double strand brake (DSB) sites, further demonstrating that ARID1A is required to create the proper chromatin profile that facilitates the histone variant H2AX phosphorylation, mediated by ATR (23).

Considering the literature findings discussed so far, we can conclude that the role of ARID1A is fundamental in different cellular processes by regulating the transcription of genes involved in cell differentiation and development, although many mechanisms are not completely clarified yet. Several recent studies reported that *ARID1A* genetic alterations are linked to tumor development. For this reason, we examined the literature describing *ARID1A* alterations in human cancer, highlighting its double role as a tumor suppressor or oncogene in different tumor types and stages and focusing on its current use in cancer therapy.

### The role of *ARID1A* in cancer

One of the most interesting results from the -omics characterization of human cancers was the discovery that chromatin regulation and epigenetic processes are tightly linked to the development of cancer (29). Indeed, nearly all cancers display epigenetic changes that alter DNA expression and chromatin accessibility, and most cancer mutations directly or indirectly affect the epigenome (30, 31). Unexpectedly, many epigenetic-related genetic alterations were attributable to genes encoding subunits of the mammalian ATP-dependent SWI/SNF complex, especially BAF (Brg/Brahma-associated factors) complex (6, 32).

**Figure 1:**
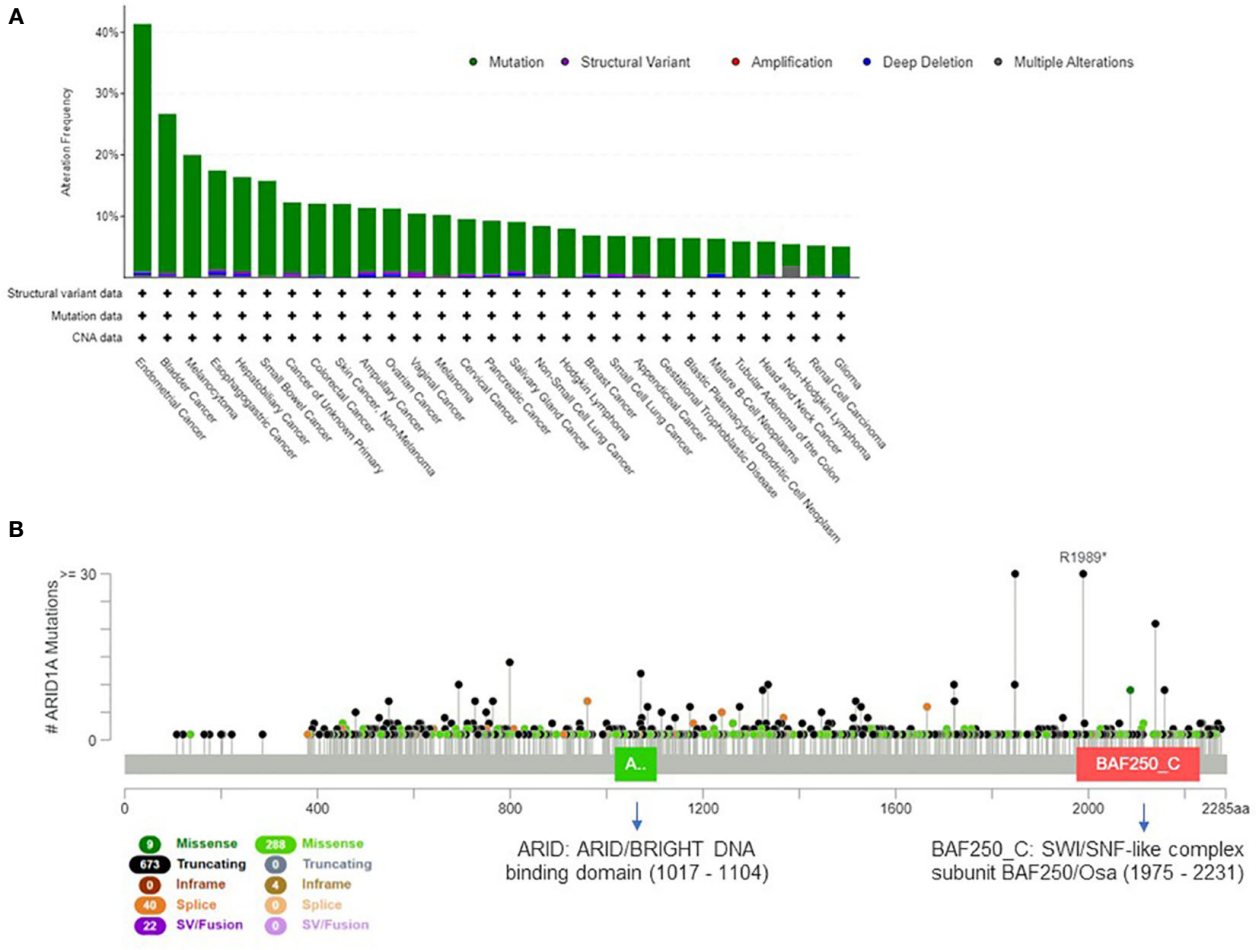
Extent of *ARID1A* involvement in human cancers. **(A)** Histogram showing *ARID1A* alteration frequency in 153.554 samples from the AACR GENIE project (39) and the alteration type across human cancers (minimum frequency cutoff at 5%). **(B)** Type, frequency and distribution of *ARID1A* mutations on the gene coding sequence across all AACR GENIE project tumor types.

The mammalian SWI/SNF complex (33) is present in multiple forms in mammalian cells, and recent studies demonstrated that the subunit combination determines the functional specificity of the enzyme (34). Overall, the 29 genes of the SWI/SNF complex are mutated in 20% of all cancer types (35); this evidence suggests that the genetic perturbation of SWI/SNF complex is critical for cancer development and can have important oncogenic implications (36, 37). The most frequently mutated gene of the complex is *ARID1A* (38), which is altered in about 10% of human cancers. In **Figure 1A** we present the tumor types with frequent *ARID1A* alterations, according to AACR GENIE project data (39). Genetic alterations are evenly distributed across the gene and comprise missense or truncating mutations that are associated with the loss of function of the protein (**Figure 1B**).

Some studies report that ARID1A exerts cancer initiation and progression activities in specific cancer types, generally solid tumors, but its function as tumor suppressor or oncogene remains an open question. Mutations in *ARID1A* gene are usually responsible for its loss of function, thus suggesting a major tumor suppressive role. The survival analysis of *ARID1A*mut *vs*. *ARID1A*wt tumors across TCGA pan-cancer studies and MSK-IMPACT cohorts queried in cBioPortal website (40), generated partially discordant results, and specifically favorable prognosis is reported for ARID1A mutant TCGA cohort (q-value<0.001 for progression-free and disease-free survival, q-value 0.1 for overall survival) and a negative prognosis (p-value 0.007) for the MSK cohort.

In light of the above evidence, ARID1A seems to have a complex role in tumor development, and more studies are required to shed light on ARID1A tumor-specific activity. So far the assessment of its function as a tumor suppressor gene or an oncogene in cancer remains an open question (41, 42).

### Ovarian and endometrial cancer

*ARID1A* mutations/deletions are documented in up to 80% of clear cell ovarian cancer (CCC), 56% of uterine endometrioid cancer (EC) (43), 40% of endometrial carcinoma and endometroid ovarian cancer and 30% of mucinous ovarian cancer (44), but in 0% of high-grade serous ovarian cancer (44, 45). Wiegand et al. specifically found that 73% of heterozygous *ARID1A* mutated tumors show a loss of protein expression without loss of heterozygosity, suggesting a haploinsufficiency mechanism (45). In addition, *ARID1A* mutations were commonly found during the early stages of endometriosis-associated ovarian carcinomas development, thus suggesting a trigger role for ARID1A loss (46). *ARID1A* mutation seems to be an early event also in endometrial glandular epithelium malignant transformation and ARID1A loss was found in areas with atypical endometriosis (47). These data suggest that ARID1A could be considered a tumor-suppressor gene in ovarian and endometrial cancers.

Gibson et al. analyzed the genomic landscape of endometrial cancer progression and reported the presence of *ARID1A* mutations since the early stages of tumor development (48). Among the driver genetic alterations, they found mutations in *PIK3CA, PTEN, TP53*, and *PPP2R1A*. *ARID1A* mutations were found to be heterogeneous and subclonal at the early stages, but related to a homogeneous ARID1A protein loss in advanced lesions (48). Reviewing all endometrial cancers in cBioPortal database (40), we observed that *ARID1A* mutations are mutually exclusive with *TP53* mutations and co-occur with *PTEN* mutations (q-value<0.001).

Bitler et al. demonstrated that ARID1A inactivation upregulates HDAC6 expression, which in turn deacetylates Lys120 of P53 (49). P53K120 acetylation is a pro-apoptotic post-translational modification that selectively regulates apoptosis, without affecting cell cycle regulation (50). Therefore, *ARID1A* mutations contribute to the final inactivation of the apoptosis-promoting function of P53 by suppressing apoptosis-promoting P53K120Ac. This finding suggests that either the transcriptional repression of oncogenic genes or the transcriptional activation of tumor suppressor genes contribute to the tumor suppressive role of ARID1A.

Unexpectedly, ARID1A inactivation in association with APC and PTEN absence in mouse ovarian cancer models, prompted tumor cells towards epithelial differentiation (51). This observation suggests a context-dependent role for ARID1A in ovarian/endometrial cancer.

### Hepatocellular carcinoma

Aberrant SWI/SNF mediated chromatin-remodeling can sustain the activity of both oncogenic and tumor suppressive networks, resulting in directionally opposite effects. A double functional role for ARID1A in tumorigenesis has been described in hepatocellular carcinoma (HCC). Indeed, a recent finding by Sun et al. demonstrated that the gain of ARID1A function triggers tumor initiation by enhancing CYP450-mediated oxidative stress, while the loss of ARID1A during the later phases of tumor growth decreases the DNA accessibility and inhibits the transcription of genes associated with migration, invasion, and metastasis (42). In this model, *ARID1A* haploinsufficiency is enough to drive tumor progression.

**Figure 2:**
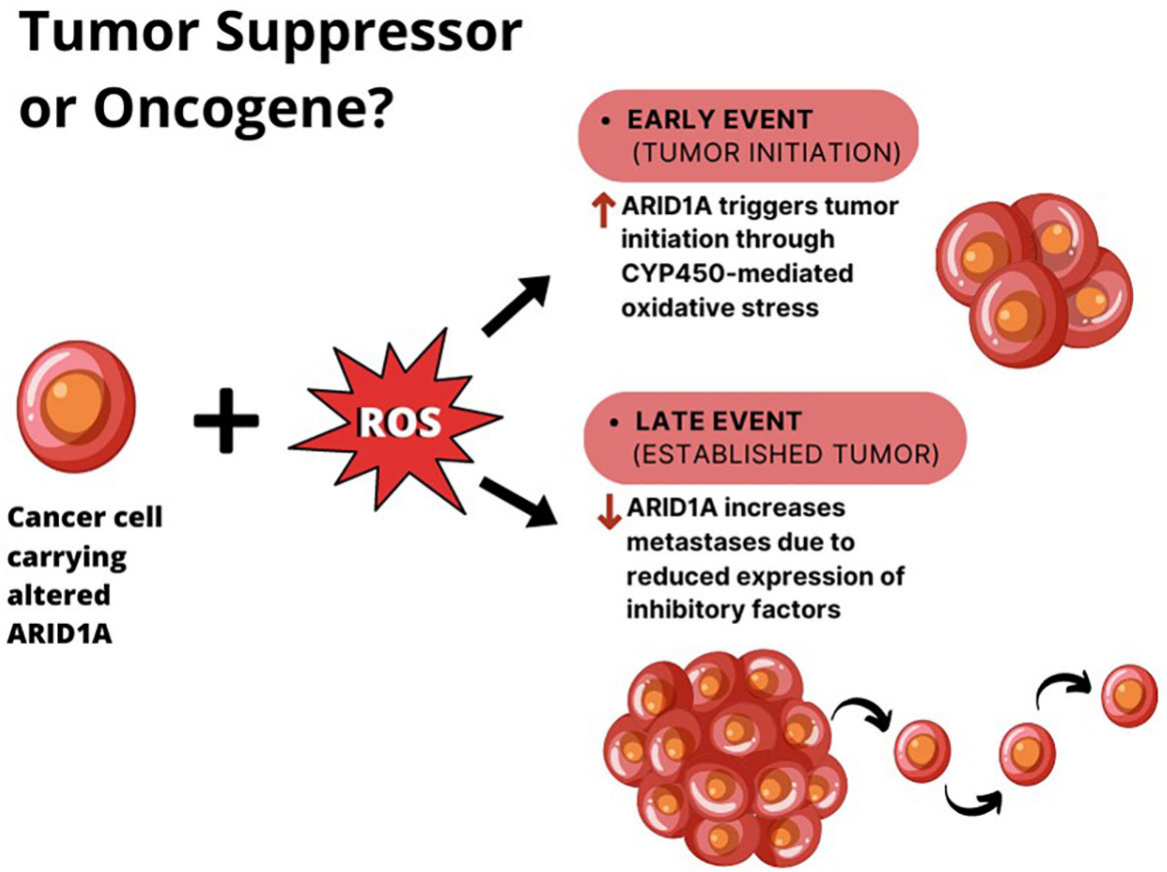
The double role of ARID1A in HCC. In the context of liver cell exposure to reactive oxygen species (ROS), ARID1A is overexpressed in cancer cells during tumor initiation, where it enhances tumor proliferation. When the tumor is established, ARID1A downregulation seems to elicit tumor metastases.

Zhao et al. reported that 10–15% of HCCs harbor loss-of-function mutations in this gene and that 83% of HCC show ARID1A mRNA overexpression if compared to adjacent normal tissues (52). According to these observations, the authors proposed two different explanations for ARID1A role in HCC (**Figure 2**):

1. the change in ARID1A expression could be an early event during the development of HCC, since the silencing of ARID1A enhances cellular proliferation. However, this hypothesis cannot explain the up regulation of ARID1A in most HCC tumors compared with adjacent normal liver tissues.
2. the expression of ARID1A is very low in normal tissues, and at the early stages of the tumorigenesis ARID1A increases to prevent cellular proliferation. Whereas, during the late stages of HCC progression, the ARID1A loss due to acquired mutations, elicits tumor escape and enhance cell proliferation.

The second hypothesis seems to be more reasonable if we consider that ARID1A expression levels negatively correlated with survival in HCC patients (53).

To test the functional role of ARID1A, Zhang and colleagues used the hepatocellular carcinoma model induced by hydrodynamic transfection of tumor cells with activated AKT/NRAS combined with either ARID1A overexpression or knockdown (54). They observed that ARID1A depletion resulted in accelerated tumor growth and decreased survival *in vivo*, while ARID1A overexpression had the opposite effect, increasing survival and slowing tumor growth.

### Gastric cancer

Gastric carcinoma (GC) is classified by The Cancer Genome Atlas (TCGA) project into four molecular subtypes: Epstein-Barr virus (EBV) positive with extreme DNA hypermethylation; microsatellite instability (MSI); genomically stable (GS), and chromosomal instability (CIN) (55).

*ARID1A* genetic alterations in gastric cancer (GC) were first reported by Abe et al. and Wang et al. (56, 57). The authors investigated the role of ARID1A loss in the context of EBV infection and genomic instability. In EBV+ GCs with microsatellite instability (or MHL1-lost), Abe et al. observed a frequent (34%) ARID1A loss of function by using immunohistochemistry (IHC) staining. In the MLH1-lost subgroup, ARID1A loss occurs in the early stages of tumor development, but only in EBV- and MLH1+ GCs the loss of ARID1A expression was associated with prognostic features. The loss of ARID1A expression is a consequence of *ARID1A* mutations (56, 58), but early stage ARID1A mutations are not always associated with loss of protein. The authors hypothesized that epithelial cells with ARID1A loss can be more easily infected by EBV and this could lately trigger cancer development (56). The transfection of ARID1A gene in gastric cancer cell lines reduced cell proliferation while ARID1A silencing promoted proliferation and migration, thus confirming ARID1A tumor suppressive role in gastric cancer (57).

A systematic meta-analysis of fourteen studies demonstrated that the loss of ARID1A expression predicts poor overall survival in gastric cancer, specifically in Asian populations suggesting a potential role as prognostic biomarker (59). Fitzmaurice et al. showed that PD-L1 is overexpressed in gastric cancer lacking ARID1A expression (60, 61). Hence, gastric cancer lacking ARID1A expression may be more sensitive to PD-1-PD-L1 immune checkpoint therapies.

### Breast cancer

*ARID1A* mutations are found in 4% of breast cancers (BCs). *ARID1A* copy number loss is the most frequent genetic alteration and involves 13–35% of BC cases. In addition, Zhang et al. demonstrated that in a variety of primary invasive BCs, ARID1A expression was epigenetically regulated. (62). Indeed in 86.4% of invasive ductal breast cancers, ARID1A low expression was related to gene promoter hypermethylation (63). Mamo et al. reported a correlation between the absent or decreased expression of the gene and increased tumor aggressiveness (64). Moreover, ARID1A protein expression was demonstrated to be an independent prognostic factor in breast cancer, with higher expression associated with better prognosis (65). Similarly, Takao et al. found that in patients with invasive breast cancer, the partial loss of ARID1A immunoreactivity was associated to a worse prognosis (66).

Among the genes interacting with ARID1A, RAB11FIP1 is overexpressed in breast cancer (67) RAB11FIP is involved in the Rab-11 mediated vesicle recycling, endosomal trafficking and transport between the recycling endosome and the trans-Golgi network, including the trafficking of integrin α5β1, required for cancer cell invasion, metastasis, and resistance to anticancer drugs. In Takao’s study, they found that the downregulation of ARID1A increases RAB11FIP1 expression, resulting in accumulation of integrin α5β1 on breast cancer cell membrane, thus enhancing cancer cell invasion. Specifically, ARID1A decrease alters the three-dimensional structure of the RAB11FIP1 promoter region thus increasing its expression and facilitating invasive breast cancer.

### Pancreatic cancer

Recent sequencing analyses of PDAC have revealed *ARID1A* mutations in 6% of the cases (68). PDAC is one of those tumors yet poorly understood. Significant recurrent mutations are found *in KRAS, TP53, CDKN2A, SMAD4, RNF43, ARID1A, TGFβR2, GNAS, RREB1* and *PBRM1*. These mutations are associated with amplification of *GATA6* (18q11.2), *ERBB2* (17q12), *KRAS* (12p12.1), *AKT2* (19q13) and *MYC* (8q24.2), and deletion of *CDKN2A* (9p21.3), *SMAD4* (18q21.2), *ARID1A* (1p36.11) and *PTEN* (10q23.31) (69).

Birnbaum et al. demonstrated that nine out of ten *ARID1A* mutated pancreatic cancers carry KRAS hotspot mutation G12D, suggesting that the inactivation of ARID1A may cooperate with KRAS in the early stages of pancreatic cancer formation (70). Li et al. also found that ARID1A deficiency, together with *KRAS*-G12D mutation, drive the development of pancreatic cancer *via* miR-503/CDKN2A axis-mediated senescence, although how ARID1A affects miR-503 transcription is not clear (71). These studies prove that ARID1A genetic alteration alone cannot initiate pancreatic cancer but can synergize with other altered genes to promote its pathogenesis.

In the COSMIC database, well-differentiated pancreatic neuroendocrine tumors (with a Ki-67 proliferation rate less than 3%) carry about 20% *ARID1A* mutations, much higher than 5.35% aggressive PDAC (72).

### Renal cell carcinoma

ARID1A acts as a tumor suppressor gene in renal cell carcinoma (RCC). To define the effects of ARID1A in renal carcinogenesis, Somsuan et al. used a non-malignant kidney epithelial (MDCK) cell line to demonstrate that ARID1A silencing using siRNAs significantly reduced cell death while increasing cell proliferation, with a cell cycle shift from G_0_/G_1_ to G_2_/M phase. In this study, they proved that ARID1A knockdown or deficiency was associated with decreased apoptosis and increased cell proliferation (73). Moreover, the siARID1A-transfected MDCK cells had higher migratory activity and invasive capability, also showing an enlargement of nuclei and multicellular spheroids.

Another study in patients affected by RCC, revealed lower ARID1A protein expression in 67% of samples and decreased ARID1A messenger RNA (mRNA) levels in 68% of samples if compared to normal kidney (74). The loss of ARID1A expression was associated with a larger tumor size, nuclear grade, and higher stage. Furthermore, ARID1A-positive cancers exhibited a longer disease-free and overall survival. Accordingly, Park et al. assessed the clinicopathological correlation and prognostic significance of ARID1A expression by an immunohistochemical study: they proved that low level of ARID1A was significantly correlated with higher nuclear grade, advanced pTNM stage, and shorter cancer-specific and progression-free survival. They proposed ARID1A expression as an independent prognostic factor for progression-free survival in RCC patients (75).

### Cancers of unknown primary

Cancers of unknown primary origin (CUP) comprise newly diagnosed tumors presenting as metastatic cancers, whose primary site cannot be identified after detailed standardized physical examinations, blood analyses, imaging, and immunohistochemical (IHC) testing (76). This tumor type is characterized by an *ARID1A* mutation frequency of 12-16%.

In a study by Ross et al. *ARID1A* mutations accounted for 11% of 200 archive CUPs (77). Moreover, in a recent study published by Laprovitera et al. we reported that the intratumor frequency of *ARID1A* mutation could be associated with CUP progression. Specifically, we longitudinally evaluated the variant allele frequency in circulating cell-free DNA (ccfDNA) samples of a CUP case with *ARID1A* mutations. The study reports how the fractional abundance of *ARID1A* mutation (p.R1276_) in ccfDNA decreases during the initial treatment and then increases again during disease worsening, thus suggesting a role in the expansion of the more aggressive subclones (78).

### Melanoma

Many genetic alterations occur during the development of melanoma. Thielmann et al. analyzed the clinical pathological features of 116 patients diagnosed with melanoma in association with the most common genetic alterations, including mutations in *ARID1A* gene (79). They demonstrated that *ARID1A* mutated melanomas exhibit higher tumor mutational burden (TMB). *ARID1A* mutations were evenly distributed across the gene without clustering or hotspots. Despite the increased TMB, no statistical significance was noticed in *ARID1A* mutated patients receiving targeted therapies or immune-checkpoint inhibitors for what concerns progression-free and overall survival. However, *ARID1A* mutated tumors revealed UV-induced mutation signatures, showing a higher frequency of C>T substitutions in comparison with *ARID1A* wild-type melanomas. This finding suggests that the impact of *ARID1A* mutations in immune-checkpoint inhibitors response needs to be better elucidated.

### Colorectal cancer

ARID1A expression is progressively lost during colorectal cancer (CRC) development: Wei et al. showed that the loss of ARID1A expression was associated to distant metastasis and late TNM stage of CRC. However, the survival analyses indicated that the loss of ARID1A protein expression was a better prognostic factor for stage IV CRC (80). In line with this observation, Erfani et al. reported no significant association between overall survival and loss of ARID1A expression in CRC (81). Other studies did not find any significant association between loss of ARID1A expression and overall survival; still, they observed that the overall survival was better for patients with no/low ARID1A expression than those with ARID1A expression (82–84). Erfani et al. demonstrated that ARID1A expression is reduced by promoter hypermethylation in CRC and its low expression is associated with lymphatic invasion. These findings suggest that the role of ARID1A in CRC is not completely understood, and possibly different than in other cancer types.

### Lung cancer

*ARID1A* mutations are detectable in about 6-7.5% of lung cancers. Hung et al. demonstrated that *ARID1A* loss-of-function mutations and biallelic inactivation induce the complete loss of ARID1A expression in non-small cell lung cancer (NSCLC) (85). Another study reported that patients with ARID1A loss had a shorter cancer specific survival and a significant association of ARID1A loss to male sex, larger tumor size, smoker status and squamous histology (86). Moreover, ARID1A-loss lung cancers had the worst survival in comparison to ARID1A-positive tumors. Thus, the loss of ARID1A expression might be a valuable prognostic marker in NSCLC (57, 63, 74).

In addition to survival rate, other clinicopathological factors such as lymph node metastasis and tumor infiltration have been positively correlated with loss of ARID1A expression (87). The authors also experimentally verified the impact of ARID1A silencing in lung cancer cell lines, concluding that ARID1A has a tumor suppressive role in this tumor type.

## ARID1A pharmacological targeting

The high frequency of *ARID1A* mutations among different cancer types, made this gene a very appealing research object for target therapy investigations.

Specifically, as a “care-taker” and “gate-keeper” gene, the mutational status of *ARID1A* in target therapy was investigated within the context of synthetic lethality. Synthetic lethality is based on essential gene interactions where a genetic alteration, such as a defect in a tumor suppressor gene (genetic context), influence a second gene essential for cell survival (pharmacological target) (88). The use of synthetic lethality as a guidance to develop cancer therapeutics was introduced by Hartwell (89) and Kaelin (90), after the success of PARP inhibitors in BRCA-mutant ovarian cancers (91–93). As for BRCA mutation, *ARID1A* deficiency in tumors constitutes a promising synthetic lethal phenotype (94) for the use of small inhibitors targeting DNA damage response (DDR), immune-checkpoint blockade (ICB), kinases, and agents leading to a cell-specific cytotoxicity.

Currently there are 23 clinical trials registered in the clinical trials website (https://clinicaltrials.gov/) concerning ARID1A, ranging from phase I to phase II. **Table 1** reports a list of concluded clinical trials where ARID1A mutation was considered in outcome evaluations. Since ARID1A deficiency was firstly discovered in gynecological cancers (95); (47, 45), many clinical trials involved uterine/ovarian cancer patients. However, other non-gynecologic clinical trials have been recruiting patients with a wide range of oncologic diseases: bladder cancer, cholangiocarcinoma, pancreatic, colorectal, biliary tract cancer, NSCLC and other solid tumors. In all these clinical trials, ARID1A was considered for synthetic lethal drug screening. The pharmacological targets include molecules directed toward DNA damage response (DDR), immune checkpoint blockade (ICB), kinases, epigenetics effectors.

**Table 1:** Completed clinical trials involving cancers with ARID1A deficiency*.

Target	Clinical trial ID	Study title	Drug (target)	Disease	Study phase	ARID1-RELATED RESULTS	Ref.
DDR, ICB and combination	NCT02506816	Preoperative Olaparib Endometrial Carcinoma Study (POLEN)	Olaparib (PARP inhibitor)	Endometrial Carcinoma	0	Patients with ARID1A deficiency had a marked effect of reducing the expression of PARP-1 and cyclin-D following Olaparib treatment.	(24)
DDR, ICB and combination	NCT02278250	First in human study of M4344 in participants with advanced solid tumors	M4344 (ATR inhibitor)	Advanced solid tumors	1	Clinical Trial results are under evaluation. M4344 showed an *in-vitro* and *in-vivo* anticancer activity through the induction of mitotic catastrophe and DNA damage. This activity is significantly correlated with ARID1A deficiency.	(25)
DDR, ICB and combination	NCT03718091	M6620 (VX-970) in selected solid tumors	M6620 (ATR inhibitor)	Solid tumor Leiomyosarcoma Osteosarcoma	2	Among the enrolled cohort of patients, authors reported the case of a patient with metastatic colon cancer harboring an ARID1A deficiency who had a complete response to therapy (progression-free survival of 29 months at the last evaluation)	(26)
Epigenetics, ICB and combination	NCT03297424	A Study of PLX2853 in Advanced Malignancies	PLX2853 (BET inhibitor)	Advanced malignancies	1,2	This study reported an encouraging pharmacological activity of PLX2853, but results comparing synthetic lethality with ARID1A deficiency are yet to be presented.	(27)
Kinases	NCT01914510	A study of ENMD-2076 in ovarian clear cell cancers	ENMD-2076 (Multikinase inhibitor)	Ovarian clear cell carcinoma	2	Although primary endpoint of the trial failed, ENMD-2076 showed an improved outcome in ARID1A deficient ovarian clear cell carcinomas patients.	(28)
Epigenetics, ICB and combination	NCT04493619	PLX2853 as a Single Agent in Advanced Gynecological Malignancies and in Combination With Carboplatin in Platinum-Resistant Epithelial Ovarian Cancer	PLX2853 (BET inhibitor) Carboplatin	Gynecologic Neoplasms	1,2	No result published yet	

*Information on the clinical trials was obtained from https://clinicaltrials.gov on December 2022. DDR, DNA- damage repair; PARP, poly(ADP- ribose) polymerase; ICB, immune check‐point blockade; ATR, ATM and rad3-related; BET, bromodomain and extra- terminal motif.

Genome stability, which essential for cell survival, is compromised in ARID1A-deficient cancer cells. As a result, cancer cells with high levels of replicative stress, become more dependent on compensatory mechanisms such as the activation of ATR signaling (96). This dependency underlies the mechanism of synthetic lethality of ATR inhibitors (ATRi)(97). There are six registered clinical trials using ATRis in patients with ARID1A-deficient solid tumors. The highly potent ATR inhibitor, M4344 (VX-803) is the pharmacological compound used in phase I clinical trial number NCT02278250, which is now concluded, and whose results are under evaluation. M4344 compound demonstrated an anticancer activity both *in-vitro* and *in-vivo* by inducing mitotic catastrophe and DNA damage: this effect was significantly correlated with *ARID1A* deficiency (25). A very interesting result about the synthetic lethality with ATRi in ARID1A-deficient tumors comes from study NCT03718091. Among the results of this study, a patient with metastatic colon cancer harboring an ARID1A deficiency had a complete response to therapy (progression-free survival of 29 months at the last evaluation) (98).

Another synthetic lethality relationship currently exploited is between DNA damage response (DDR) deficiency and PARP inhibitors. In this context Shen et al. demonstrated both *in-vitro* and *in-vivo* that PARP inhibitors are selectively active towards ARID1A-deficient cells, thus providing a novel approach for stratifying patients for clinical trials of targeted therapy with PARP inhibitors (23). The POLEN study was a window-of-opportunity trial where PARP inhibitor Olaparib was administered as neoadjuvant therapy in patients with early-stage endometrial carcinoma before surgery (24). Authors showed that treatment with Olaparib reduced the expression of PARP-1 and cyclin-D, this effect was more prominent in patients with ARID1A deficiency.

Another PARP inhibitor, niraparib, was used in a phase II clinical trial in metastatic solid tumors (99). Patients were stratified for the presence of mutations in genes involved in DNA damage repair including *ARID1A* (court B). Preliminary results of the study were presented at ASCO 2022 and described that in the court B patients who had stable disease were *ARID1A* mutated (99). Moreover, there are preclinical studies associating the loss of ARID1A function with synthetic lethality based on inhibitors of the bromodomain and extra-terminal family, BET (BETi) (100); (16, 101). This led to the development of clinical trials involving BET inhibitors as single agents or in combination with existing treatment options in multiple human cancers bearing ARID1A deficiency. On this basis, PLX2853, a potent BET inhibitor, was used in two completed phase I/II clinical trials (NCT03297424, NCT04493619). Gordon et al, presented early results from the “PLX2853 in Advanced Malignancies” study reporting an encouraging pharmacological activity, but results comparing synthetic lethality with ARID1A deficiency are yet to be present (27). Targeting Aurora kinase A (AURKA) is a further synthetic lethality interaction in ARID1A defective tumors. Starting from the finding of Wu et al. unveiling the direct repression of AURKA by ARID1A (102), studies have been conducted with pan-aurora kinase inhibitors in colon and ovarian cancer cells with *ARID1A* deficiency, causing chromosomal abnormalities leading to synthetic lethality (103).

Finally, a phase 2 clinical trial to assess the activity of a strong selective inhibitor for AURKA, ENMD- 2076, in treating patients with ovarian clear cell carcinomas was recently concluded (NCT01914510). Despite ENMD- 2076 did not meet the efficacy bar set in this trial, this AURKA inhibitor has been reported as potentially advantageous for ovarian clear cell carcinomas patients with ARID1A deficiency (28).

## Conclusions

ARID1A acts predominantly as a tumor suppressor gene in many solid tumors, although its functional role seems to be stage and tumor type dependent. For example, ARID1A expression is initially upregulated in HCC and then lost by acquisitions of truncating/missense mutations. The loss of ARID1A expression in human cancers is generally associated with negative prognostic features, tumor progression and increased tumor growth and invasion. The lack of ARID1A in tumors is associated with an increased tumor mutational burden and genomic instability, which is currently pharmacologically exploited according to synthetic lethality principles. Although the exact mechanisms are still elusive, clinical trials with small inhibitors directed toward DNA damage response (DDR), immune checkpoint blockade (ICB), kinase inhibitors and epigenetics effectors are showing promising results and are worth investigating in future studies.

## Author contributions

MF contributed to conception and design of the study. BF, IS, GG, and RR wrote the first draft of the manuscript. IP wrote sections of the manuscript. All authors contributed to manuscript revision, read, and approved the submitted version.
